# Elucidation of a Novel Dual Binding Site on Tubulin: Theoretical Insights and Prospective Hybrid Inhibitors

**DOI:** 10.3390/ph19010003

**Published:** 2025-12-19

**Authors:** Dmytro Khylyuk, Oleg M. Demchuk, Rafał Kurczab, Barbara Miroslaw, Monika Wujec

**Affiliations:** 1 Department of Organic Chemistry, Faculty of Pharmacy, Medical University, 20-093 Lublin, Poland; monika.wujec@umlub.edu.pl; 2 Faculty of Medicine, The John Paul II Catholic University of Lublin, Konstantynow 1J, 20-708 Lublin, Poland; 3 Department of Chemistry, Faculty of Mathematics and Natural Sciences, University of Applied Sciences in Tarnow, Mickiewicza 8, 33-100 Tarnow, Poland; r_kurczab@atar.edu.pl; 4 Department of General and Coordination Chemistry and Crystallography, Institute of Chemical Sciences, Faculty of Chemistry, Maria Curie-Sklodowska University in Lublin, Maria Curie-Sklodowska Sq. 2, 20-031 Lublin, Poland; barbara.miroslaw@mail.umcs.pl

**Keywords:** α-tubulin, dual binding site, pironetin, todalam, covalent inhibitor, anticancer activity

## Abstract

**Background/Objectives:** Microtubule-targeting agents remain foundational components of anticancer chemotherapy, yet their clinical utility is constrained by resistance and toxicity. **Methods:** Here, we present a theoretical exploration of a plausible “dual” binding pocket that spans the α-tubulin pironetin site and the inter-subunit todalam site. Eight virtual chimeric ligands, each merging key pharmacophoric elements of pironetin and todalam, were constructed and covalently docked to Cys316 of α-tubulin. **Results:** Covalent docking followed by 200 ns all-atom molecular dynamics simulations revealed that two derivatives (compounds **4** and **8**) stably occupy the merged cavity, simultaneously anchoring in the pironetin region via Michael addition and in the todalam region via π-stacking and hydrogen bonding. These hybrids preserved the critical hydrogen-bonding networks of both parent ligands and exhibited low ligand RMSD values (~1.5 Å) and compact radii of gyration throughout the simulations, indicating a tight, persistent binding. Estimated HYDE affinities of 1.5 µM for compound 4 and 17.6 µM for compound **8**, calculated with SeeSAR, suggest that covalent engagement can compensate for moderate non-covalent binding scores. **Conclusions:** In summary, our results provide compelling grounds for developing a new class of α-tubulin inhibitors that engage the hybrid pocket, laying a foundation for the structure-guided synthesis of first-in-class dual-site compounds capable of overcoming resistance to conventional microtubule-targeting drugs.

## 1. Introduction

Cancer remains one of the leading causes of death worldwide. It is estimated that by 2040, the number of cancer cases will increase to 28.4 million [1,2]. Despite the progress made in recent years in the field of cancer pharmacotherapy, there is still a growing need for new drugs that are more active and selective, have fewer side effects, and are more cost-effective [3,4,5]. One of the more promising approaches involves the development of novel small molecules targeting tubulin. Tubulin is a protein responsible for essential cellular processes such as mitosis, intracellular transport, and maintenance of cellular structure [6,7,8,9]. Tubulin has long been recognized as an attractive target for anticancer therapy. Since the discovery of colchicine as a tubulin polymerization inhibitor, numerous chemotherapeutic agents have been identified that modulate tubulin dynamics in various ways. Over time, the concept of distinct tubulin binding sites has emerged, classified by both their location on the tubulin heterodimer and the nature of the ligands they accommodate [10,11]. In addition to the classical colchicine-binding site, other important sites have been characterized, including the vinca, taxane, and laulimalide binding sites, all of which reside primarily in the β-subunit of tubulin or at the interface between the α- and β-subunits [12]. In contrast, binding sites located within the α-subunit—including the gatorbulin, todalam, and pironetin sites [13]—remain much less studied, despite their potential for destabilizing microtubules and inhibiting their polymerization [14].

Comprehensive analysis of binding sites in tubulin using a combination of crystallographic data and computational approaches has enabled the identification of additional novel binding site, which can be targeted by different small molecules [15]. Tubulin inhibitors have achieved success in clinical practice, but their therapeutic efficacy is often limited by rapid development of drug resistance and dose-limiting toxicity [16]. Therefore, there is growing interest in designing dual-target drugs that can simultaneously modulate multiple molecular pathways and thus may exhibit enhanced therapeutic efficacy [17,18,19]. Based on this concept, dual-target tubulin inhibitors have been developed intensively in recent years. Simultaneous inhibition of tubulin and other key targets, such as receptor tyrosine kinases, estrogen receptors, histone deacetylases, or topoisomerases, has shown significantly better antitumor activity than compounds targeting tubulin alone [20,21].

Pironetin, an α/β-unsaturated lactone, is unique among tubulin inhibitors in that it binds covalently to the α-subunit of tubulin. Unlike most known tubulin inhibitors, which typically bind non-covalently, pironetin forms a covalent bond with Cys316, distinguishing its mechanism of action from other tubulin-targeting agents [22]. Originally isolated from the fermentation broths of *Streptomyces* species, pironetin remains the only natural product known to bind α-tubulin covalently. Multiple in vivo studies have demonstrated its significant antitumor efficacy in murine models of leukemia, and it also retains activity against tumor types resistant to other microtubule-targeting drugs [23,24].

In contrast, todalam binds to a novel site within the α-subunit of tubulin, where it disrupts microtubule networks, induces cell cycle arrest at G2/M, and triggers cell death. It also displays synergistic effects in combination with vinblastine. Notably, the N-phenylacetamide core of todalam engages in π–π stacking with Trp407 and hydrogen bonding with Asn102. By acting as a molecular plug that sterically blocks the curved-to-straight conformational change in α-tubulin and by sequestering tubulin dimers into assembly-incompetent oligomers, todalam effectively destabilizes microtubules [25]. Preliminary cytotoxicity data indicate that todalam exhibits anticancer potency against HeLa and MDA-MB-231 cells.

**Figure 1:**
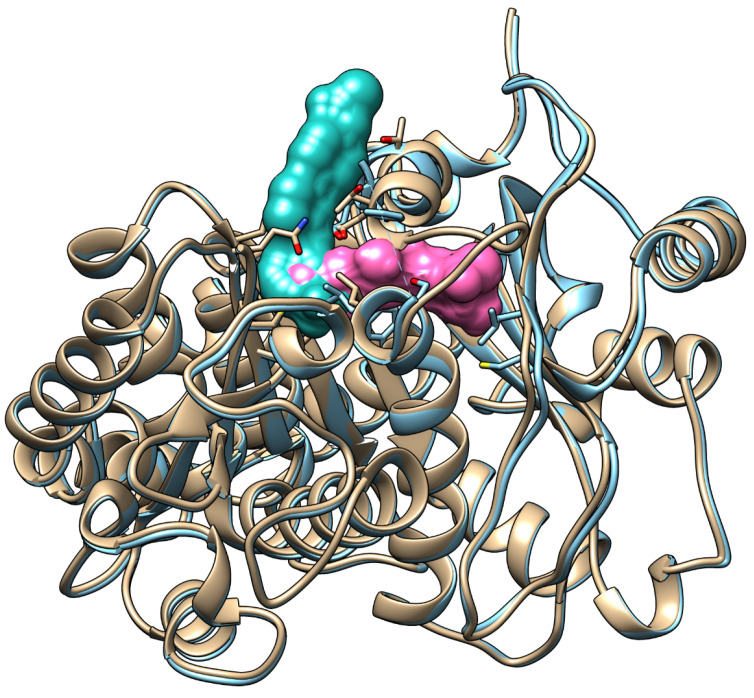
Overlay of the α-tubulin subunits co-crystallized with todalam (cyan, PDB: 5SB7) and pironetin (pink, PDB: 5LA6), revealing a shared lipophilic binding pocket occupied by both compounds.

Overlaying the α-tubulin co-crystal structures of pironetin (PDB: 5LA6) and todalam (PDB: 5SB7) reveals that both compounds occupy overlapping lipophilic pockets composed mainly of hydrophobic residues, including Cys4, Leu136, Leu167, and Leu242. The 3-CF_3_ phenyl substituent of todalam and the alkyl tail of pironetin both reside in this shared hydrophobic cavity (Figure 1).

By merging these two connected binding sites, one can view the pironetin and todalam binding regions as forming a single, expansive “dual” site. Molecules capable of engaging this entire hybrid site may induce substantial structural alterations in both subunits, thereby preventing or significantly disrupting microtubule assembly. Moreover, targeting such a large binding area could help forestall the development of resistance, analogous to how multi-target agents can impede resistance emergence in other therapeutic contexts [26,27]. The proposed dual binding site represents a structurally continuous pocket formed by the overlap of the pironetin and todalam sites, unlike classical tubulin pockets that are spatially independent. This merged cavity enables, in principle, the design of a single hybrid ligand capable of simultaneously engaging both subsites. Functionally, such dual-site binding may combine irreversible α-tubulin modification with intersubunit disruption, potentially enhancing inhibitory potency and reducing susceptibility to resistance. To date, no systematic experimental studies have addressed the biological significance of the merged pironetin–todalam cavity. Although the overlap of these sites has been noted in structural analyses, its functional implications remain unexplored. Therefore, the present study was designed to explore this hypothesis at a theoretical level as an initial step toward future experimental validation.

Herein, we present the theoretic concept of a unique strategy for developing novel tubulin polymerization inhibitors that target the understudied α-tubulin. These new inhibitors simultaneously engage two pockets of α-tubulin, anchored by a covalent bond at the pironetin site and by dispersive interactions at the todalam site. Furthermore, the location of the todalam pocket at the α/β-tubulin interface provides opportunities for ligand modification with substituents that prevent β-tubulin subunit association. At present, we have not found any studies in the scientific literature describing inhibitors that simultaneously target two different binding sites on α-tubulin. However, there are reports of compounds that show affinity for binding sites on either α- or β-tubulin [28,29]. Recognizing the scope and complexity of this endeavor, we employed contemporary in silico approaches to evaluate the feasibility of creating such hybrid ligands and to assess their binding efficacy at the α-tubulin subunit.

## 2. Results

Starting from the core scaffolds of the established tubulin inhibitors pironetin and todalam, we generated structures of eight hybrid molecules (virtual ligands) that integrate key features of both parent compounds. As shown in Figure 1, the docked α-tubulin pironetin and todalam overlap in the region of the meta-substituted phenyl ring of todalam and the propenyl fragment of pironetin. Moreover, the propenyl double bond directly overlaps with one of the C–C bonds of the aromatic system. In the corresponding crystal structures of the complexes (PDB: 5LA6) and (PDB: 5SB7), the CF_3_ group of todalam occupies an individual micropocket localized near the Cys4, while the 5-methyl group of pironetin occupies a micropocket near Leu242. The ligand structures were merged by replacing the propenyl group of pironetin with the todalam motif connected at position 2 of the terminal aryl. We design the structures to preserve positioning of the CF_3_ group at the micropocket near Cys4 and the CH_3_ group near Leu242 (ligand **4**). Since the geometry of the compounds was changed upon merging, optimal fitting was possible only when one of these substituents was present in the ligand, so the ligand **5**, without the CH_3_, and the ligand **6**, without the CF_3_ groups, were designed. The hybrid model in which CH_3_ and CF_3_ groups were incorporated into a difluorocyclohexyl ring represents ligand **7**. A more rigid structure that better reflects the shape of the combined pironetin–todalam pocket (ligand **8**) has the difluorocyclopentyl motif. Finally, additional substituents including dioxymethylene (ligand **1**), thiol (ligand **2**), and dichloro groups (ligand **3**) were designed to optimize the pironetin–todalam pocket occupancy. In the present study, eight model structures were designed to examine their ability to form stable complexes with α- and β -tubulin heterodimer.

The objective of the studies was to assess how these derivatives modulate the individual α- and β-tubulin subunits and to elucidate the inter-subunit crosstalk that emerges when these relatively bulky, chimeric inhibitors bind simultaneously to the tubulin heterodimer.

**Figure 2:**
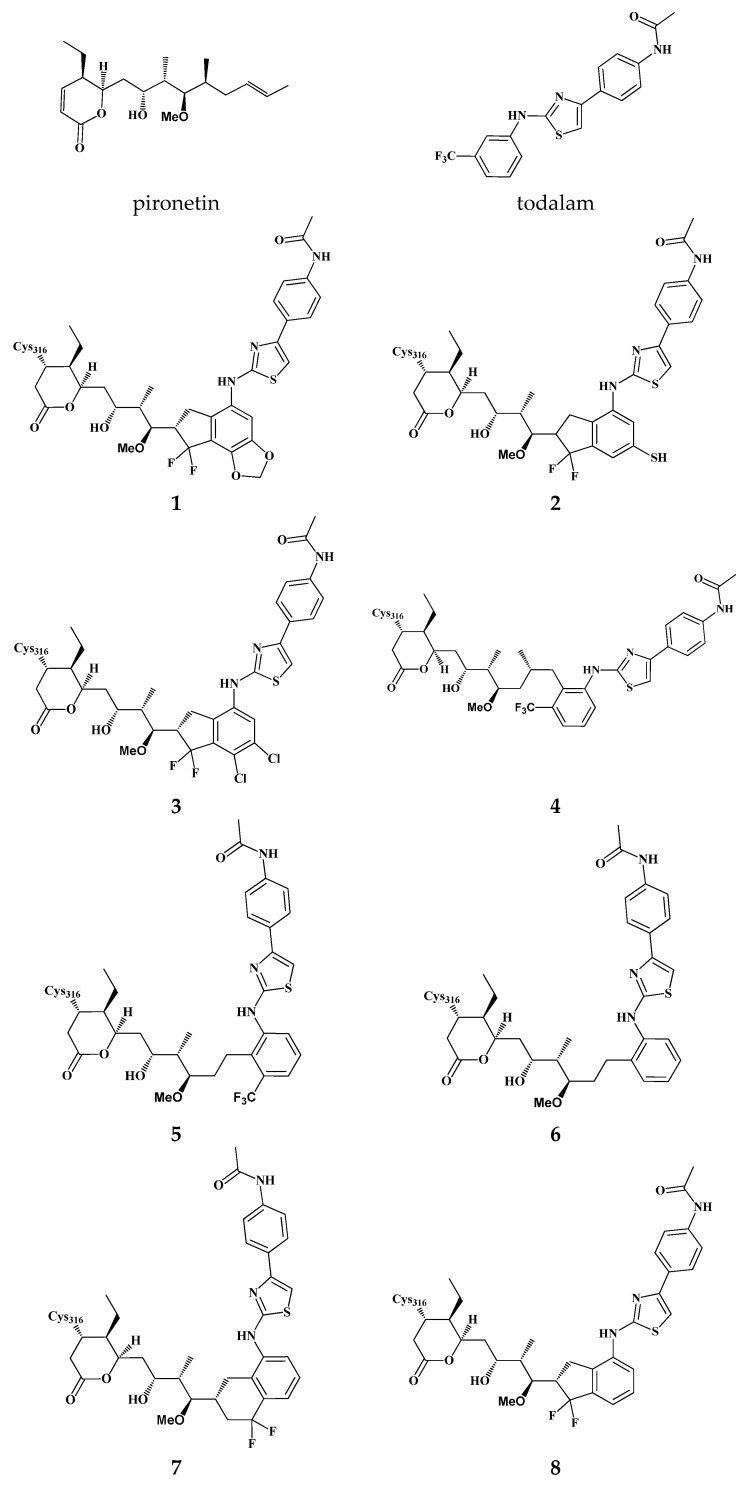
Structures of the eight designed hybrid ligands **1**–**8** created by merging key motifs from pironetin and todalam. All compounds are in silico designs and have not yet been synthesized.

Docking studies support the plausibility of the proposed hypothesis that two adjacent binding sites can function as a single novel dual binding site. The reliability of our studies was confirmed by the fact that docking of pironetin and todalam on α-tubulin returned the complexes corresponding to (PDB: 5LA6) and (PDB: 5SB7). Virtual hybrid ligands (**1**–**8**), designed by combining structural features of todalam and pironetin, demonstrated the ability to occupy spatial positions closely overlapping the original binding sites of both parent compounds (Figure 2). Ligands **4** and **8** showed the most promising results in terms of predicted affinity. However, the proposed inhibitors exhibited lower predicted binding affinities in docking studies compared to the original ligands (Table 1). These findings suggest that the hybrid binding pocket can accommodate dual-site ligands, thereby validating the structural rationale behind the design of such covalent hybrid inhibitors. Nonetheless, the primary objective at this stage was to construct virtual ligands capable of occupying the dual binding site and inducing significant structural perturbations in the 3D structure of tubulin, primarily within the α-subunit and affecting its interaction with the β-subunit.

**Figure 3:**
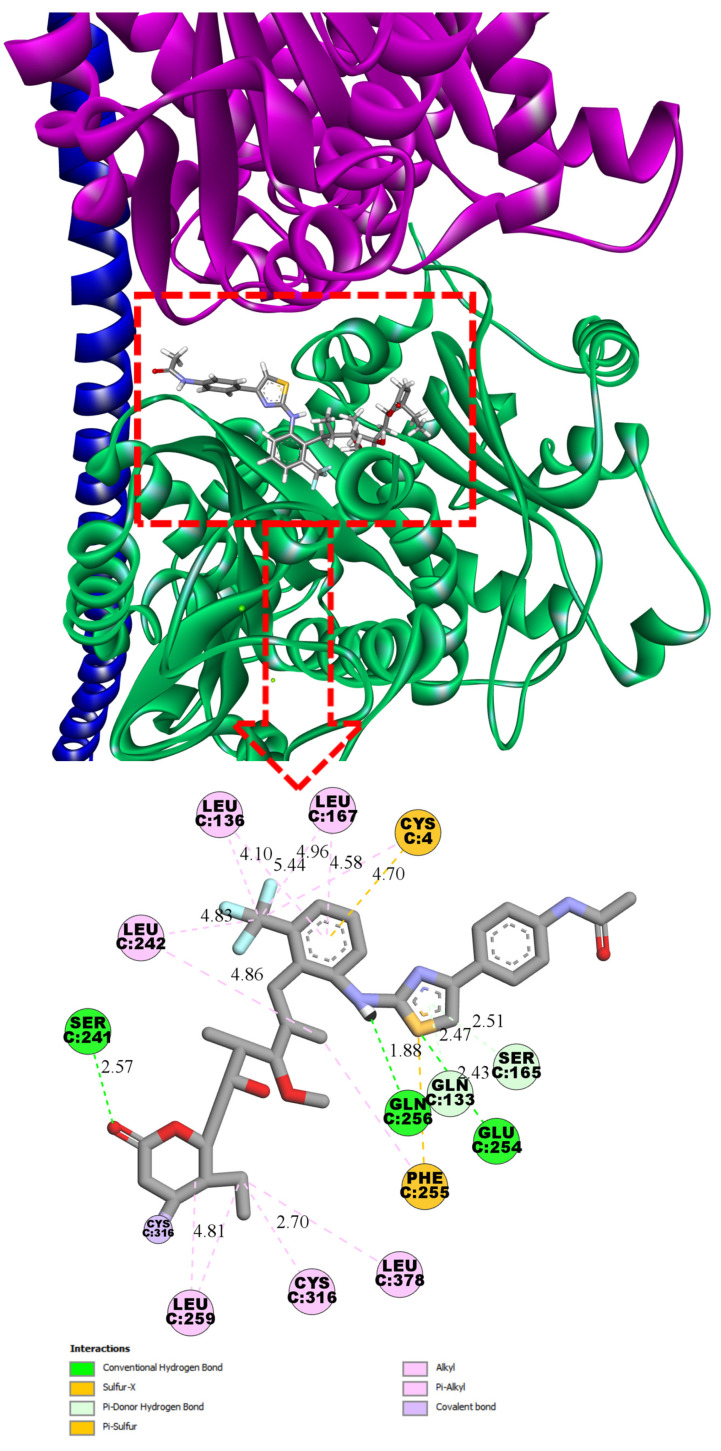
Three-dimensional and two-dimensional interaction diagrams of ligand **4** at the α/β-tubulin interface, showing the dual binding site between α-subunit (pink) and β-subunit (green) (PDB: 5LA6). The binding pose was obtained from covalent docking calculations. Predicted upper boundary affinity for compound **4** ~ 1.5 µM.

Ligand **4**, in its most energetically favorable conformation, forms hydrogen bonds with Ser241 (2.57 Å), Gln256 (1.88 Å), and Glu254 (2.43 Å). The trifluoromethylphenyl moiety occupies a characteristic lipophilic pocket shared with both todalam and pironetin, establishing a series of non-covalent lipophilic interactions, including alkyl, π-alkyl, and π-sulfur contacts. The phenylacetamide tail deviates from the orientation observed in todalam and does not participate in any specific interactions (Figure 3).

**Figure 4:**
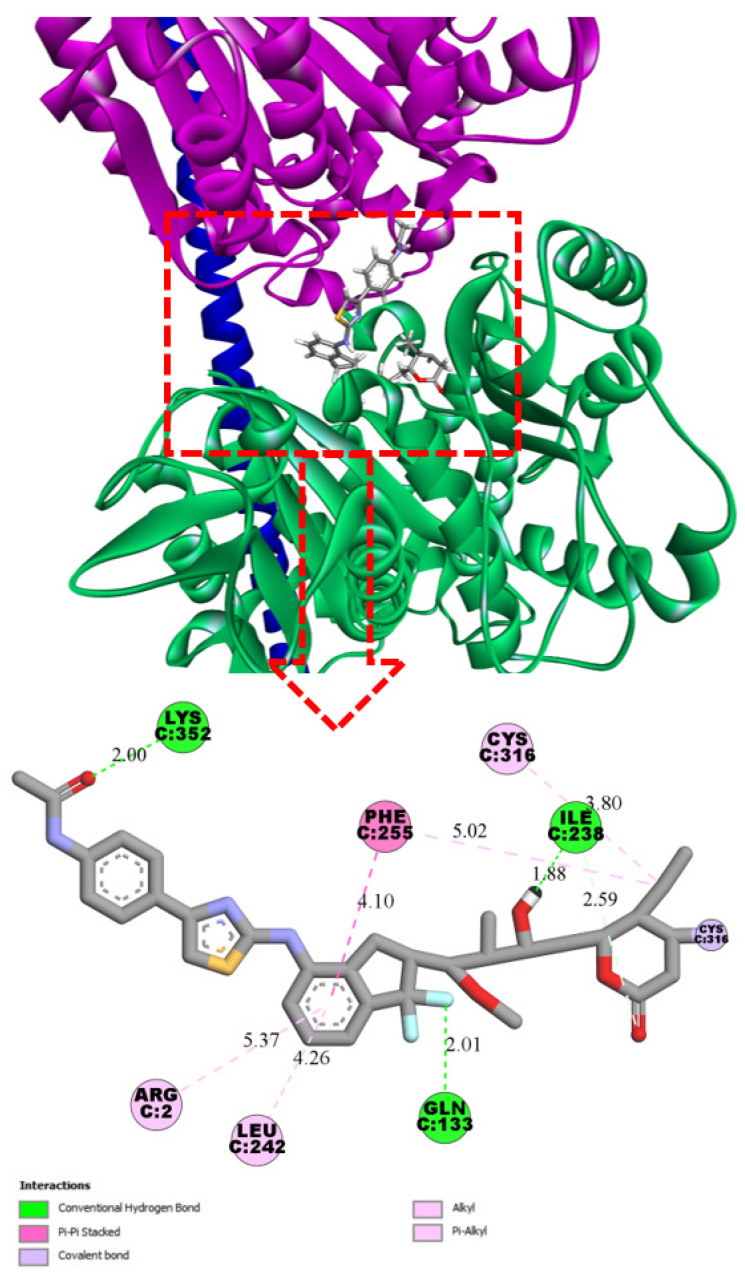
Three-dimensional and two-dimensional interaction diagrams of ligand **8** at the α/β-tubulin interface, showing the dual binding site between α-subunit (pink) and β-subunit (green) (PDB: 5LA6). The binding pose was obtained from covalent docking calculations. Predicted upper boundary affinity for compound **8** ~ 17.6 µM.

Ligand **8** also forms three hydrogen bonds: with Lys352 (2.00 Å), Ile238 (1.88 Å), and Gln133 (2.01 Å). In contrast to **4**, ligand **8** engages its phenylacetamide moiety in additional interactions. However, within the binding site shared by todalam and pironetin, the lipophilic interactions are less pronounced and involve fewer hydrophobic amino acid residues. As a result, the overall binding affinity of **8** is lower than that of **4** (Figure 4).

To investigate the binding stability and conformational behavior of the proposed hybrid ligands (**4**–**8**) targeting the pironetin and todalam sites within the tubulin structure, 200 ns molecular dynamics (MD) simulations were performed. The simulation system comprised the α/β-tubulin heterodimer in order to provide the appropriate local environment for the binding sites of todalam and pironetin, while excluding large-scale polymerization and depolymerization events that occur at the microtubule level. The selected simulation time of 200 ns is sufficient to capture local conformational changes within the binding pocket and to monitor persistent interactions with the proposed hybrid ligands. While longer simulations (µs timescale) would be required to probe dynamic instability and global rearrangements of tubulin polymers, such processes were beyond the scope of the present study. Our focus was strictly on local binding stability and structural adaptation within the ligand-binding pocket.

**Figure 5:**
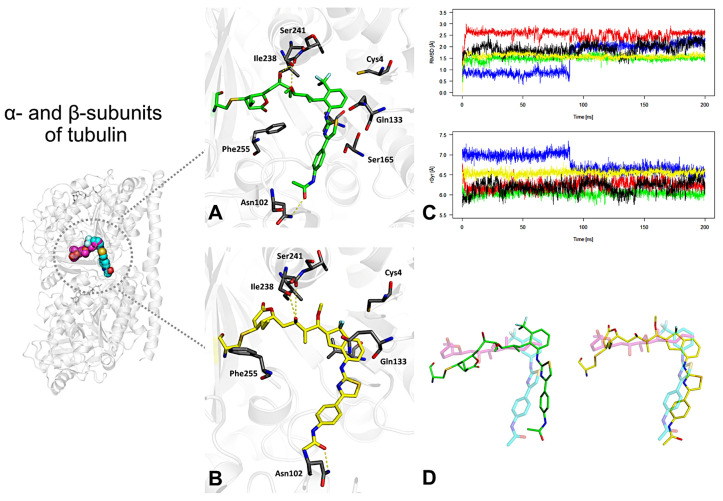
Results of molecular dynamics (MD) simulations for five proposed hybrid tubulin inhibitors designed to verify the hypothesis of simultaneous binding at the pironetin and todalam sites. (**A**,**B**) Representative binding poses of **4** and **8** in the binding site, obtained from clustering of 200 ns MD trajectories. (**C**) RMSD and radius of gyration (rGyr) profiles of **4** and **8** during 200 ns MD simulations, indicating overall conformational stability. Colors: **4** (green), **5** (red), **6** (blue), **7** (black), **8** (yellow). (**D**) Superposition of the MD-derived geometries of **4** and **8** with the native conformations of pironetin (magenta) and todalam (cyan).

The starting conformations were obtained from covalent docking procedures, which positioned the ligands within the tubulin binding interface. The MD simulations aimed to identify ligands with favorable stability profiles and explore the topological features of the newly defined hybrid binding pocket. Among the studied derivatives, compounds **4** and **8** exhibited the highest structural stability throughout the simulation time. Representative complex structures, obtained through clustering of MD trajectories, are presented in Figure 5A,B for **4** and **8**, respectively. These geometries correspond to the most populated conformational states sampled during the simulation. Interaction analysis revealed stable hydrogen bonds with residues Ile238, Ser241, and Asn102, which likely contribute to the favorable binding of these compounds. Additionally, stabilizing CH–π/π–π interactions were identified with Phe255 and Gln133, further supporting the affinity of these ligands for the hybrid pocket.

The stability of the ligand–receptor complexes was quantitatively assessed using RMSD and radius of gyration (rGyr) profiles, as shown in Figure 5C. Ligands **4** (green line) and **8** (yellow line) maintained the lowest RMSD values, with mean deviations around 1.5 Å, indicating minimal conformational drift after the initial geometry relaxation. The rGyr values were consistent with compact and stable ligand geometries: compound **4** displayed the lowest average rGyr (6.0 Å, SD = 0.07), while compound **8** showed intermediate values (6.5 Å, SD = 0.06) but with the least fluctuation during the trajectory, suggesting a well-adapted fit within the binding pocket.

The superposition of the MD-derived geometries of compounds **4** and **8** onto the native structures of pironetin (magenta) and todalam (cyan) is presented in Figure 5D. The overlay demonstrates a high degree of spatial and pharmacophoric alignment, supporting the hypothesis that these designed derivatives successfully mimic the key structural features of the reference ligands.

Because the investigated ligands form a covalent bond with Cys316 of α-tubulin, the positional fluctuations of the ligand itself are inherently restricted, rendering ligand-only RMSD a less informative descriptor of complex stability. In such systems, the ligand remains firmly anchored within the binding site throughout the trajectory, and its apparent stability is largely determined by the rigidity imposed by the covalent linkage. Therefore, our analysis focused on the overall conformational response of tubulin, as reflected by global RMSD and local rearrangements within the binding pocket. These parameters provide a more meaningful indication of how dual-site covalent inhibitors can induce structural adaptation and potential destabilization of the α/β-tubulin interface.

## 3. Discussion

Our in silico study supports the concept of a hybrid tubulin pocket created by merging the pironetin site on α-tubulin with the todalam site at the α/β-tubulin interface. Docking indicated that several designed ligands can span this combined cavity, validating its structural continuity and druggability. Ligands **4** and **8** were the top performers: compound **4** showed the lowest predicted non-covalent affinity (~1.5 µM) and formed three hydrogen bonds plus extensive hydrophobic contacts across both subunits, whereas **8** engaged a complementary set of interactions with slightly weaker affinity.

In this work, we hypothesize that a single small molecule can engage an extended and shared binding region, thereby exerting a multi-target mode of action within one chemical entity. This type of binding mode is increasingly recognized as a rational strategy to reduce the likelihood of resistance development, as it requires the simultaneous accumulation of multiple compensatory mutations in the target(s) to maintain viability. Structural and evolutionary studies reported in the literature demonstrate that ligands interacting with conserved or overlapping functional regions tend to impose a higher genetic barrier to resistance compared to single-site inhibitors. Accordingly, compounds that span extended binding pockets or interact with multiple subsites have been shown to delay or prevent resistance emergence in several biological systems [30,31,32]. The lower predicted affinities of the hybrid compounds compared to the parent ligands can also be rationalized structurally. When the pharmacophoric fragments of pironetin and todalam are merged into a single scaffold, the resulting hybrid cannot fully reproduce the optimal binding geometry of either parent molecule. The compromise in conformational fit and local orientation within each subsite likely reduces the non-covalent contribution to binding energy. Nevertheless, covalent anchoring to Cys316 and the ability to engage residues across both subpockets compensate for this partial loss of complementarity, resulting in stable complexes during molecular dynamics simulations. The potential off-target reactivity of covalent warheads was considered during design. The proposed inhibitors employ an α,β-unsaturated lactone, a relatively weak Michael acceptor that typically requires precise binding-site alignment for reaction, thereby limiting nonspecific cysteine modification. Importantly, pironetin utilizes the same electrophilic motif and has been shown to selectively modify α-tubulin in cells, supporting the feasibility of achieving site-specific covalent engagement with this class of warheads [33,34,35]. The optimal binding pose was selected based on the HYDE upper-bound score, chemically valid covalent geometry with Cys316, absence of steric clashes, and consistent interactions within both the pironetin and todalam subsites. For each compound, multiple poses were generated and evaluated, and the top-ranked pose was chosen based on overall structural plausibility and predicted affinity. These values do not account for the energy contribution of the covalent bond with Cys316 and therefore do not represent the full binding affinity. However, they allow for a preliminary assessment and ranking of the non-covalent contribution to interaction with the dual hybrid site. We used these estimates solely for internal ranking within the covalent ligand series and to identify certain patterns that could guide the future design of inhibitors targeting this dual binding site. Although the dynamics of tubulin as a polymer (in particular, the polymerization/depolymerization processes of microtubules) occur on microsecond and longer timescales [36], our study focused on local conformational changes within the α/β-tubulin heterodimer, particularly in the ligand-binding region (pironetin/todalam-binding site). Given this, a 200 ns trajectory duration was chosen as a widely accepted standard for evaluating binding stability, hydrogen bond formation, and RMSD fluctuations. For example, in a 200 ns MD simulation of the FtsZ protein, the system reached an RMSD plateau after approximately 50 ns [37], while a 200 ns simulation of the βT7 loop in the α/β-tubulin dimer revealed key local motions throughout the trajectory [38]. In our case, RMSD stabilization was similarly observed toward the end of the 200 ns interval, which allowed us to extract representative structures from the final portion of the trajectory.

Mechanistically, a ligand occupying the dual site could combine pironetin-like α-tubulin distortion with todalam-style intersubunit wedging, simultaneously stabilizing α-tubulin in the curved conformation and blocking longitudinal dimer addition. Such dual-action inhibition may intensify microtubule destabilization, reduce the effective dose, and complicate resistance because mutations in either subunit alone are unlikely to abolish binding. By engaging α-tubulin, these ligands could also retain activity against tumors that alter β-tubulin isotype composition, a frequent route to taxane or vinca resistance.

The design strategy presented here exemplifies a dual-site inhibition concept in which a single ligand simultaneously engages two neighboring but functionally distinct subsites within α-tubulin. By covalently anchoring at the pironetin site and extending into the todalam region through complementary non-covalent interactions, the hybrid molecules combine two mechanisms of microtubule destabilization: irreversible α-tubulin modification and steric blockade of the α/β-tubulin interface. Such dual engagement is expected to enhance binding persistence and structural perturbation of tubulin, thereby amplifying the inhibitory effect. Importantly, dual-site covalent inhibitors may reduce the likelihood of resistance, as mutations affecting a single subpocket alone are unlikely to fully abolish binding. This design paradigm extends the scope of classical tubulin-targeting agents and introduces a rational framework for the next generation of hybrid covalent inhibitors that exploit composite binding pockets to achieve superior potency and durability of action.

The proposed hybrid inhibitors may influence microtubule polymerization dynamics beyond the local α/β-tubulin interface by simultaneously engaging two spatially distinct regions of the tubulin dimer. Concurrent stabilization of α-tubulin in a curved, assembly-incompetent conformation and interaction with the pironetin binding site on β-tubulin could interfere with longitudinal and lateral tubulin–tubulin contacts within the microtubule lattice and thereby affect filament stability.

Dual-site binding is therefore expected to modulate tubulin conformational states, and this conformational control represents the intended pharmacological mechanism rather than an unintended effect. Nevertheless, as with any covalent or multivalent inhibitor, the possibility of additional structural perturbations cannot be excluded. At present, these conclusions are based on structural rationale and molecular modeling, and experimental validation will be required to assess both the magnitude and selectivity of the conformational effects. Future studies will therefore include in vitro tubulin polymerization assays, structural investigations, and molecular dynamics simulations to comprehensively evaluate the impact of these compounds on microtubule behavior and function.

We initially considered performing MM-GBSA calculations to estimate the binding affinities of the designed ligands toward α-tubulin. However, the standard MM-GBSA protocol assumes a reversible (noncovalent) ligand–receptor equilibrium, and is therefore intrinsically unsuited to a system where the inhibitor is chemically “locked” to the enzyme via a covalent bond. In a covalent complex, the total affinity arises from both the noncovalent docking step and the covalent bond-forming reaction. In fact, Awoonor-Williams and Abu-Saleh have shown for SARS-CoV-2 M^pro^ inhibitors that covalent and noncovalent ΔG contributions can differ substantially, underscoring that an MM-GBSA score (which omits the covalent reaction) fail to capture the full binding free energy [39]. Moreover, MM-PB/GBSA is generally recognized to overestimate absolute binding energies and is best used for relative ranking, neither of which remedies the fundamental point that the ligand cannot dissociate without bond cleavage [40]. For these reasons, we chose not to report the MM-GBSA ΔG values: although they were computed, the covalent bond makes those numbers essentially uninformative in the context of irreversible binding.

The exclusive reliance on computational approaches represents an inherent limitation of the present study. Although in silico methods offer valuable mechanistic insight and enable hypothesis-driven molecular design, they cannot fully reproduce the complexity of biological systems. Factors such as protein flexibility, solvent effects, intracellular environment, off-target interactions, and compound stability are only approximated and may influence predictive accuracy.

Additional limitations include the absence of experimental validation and the modest micromolar binding affinities predicted by docking. Accordingly, the conclusions should be considered hypothesis-generating rather than definitive. Future work will therefore focus on biochemical binding assays, in vitro tubulin polymerization studies, and cell-based antiproliferative evaluations, followed by medicinal chemistry optimization to achieve nanomolar potency and improved drug-like properties. Structural characterization of tubulin–ligand complexes by X-ray crystallography or cryo-EM will furthermore be essential to confirm the proposed dual-site binding mode and to guide rational refinement of these hybrid inhibitors.

The next phase of this work will therefore focus on the synthesis of the first prototype compounds, followed by experimental evaluation of ADMET properties. It should also be noted that deviations from Lipinski’s Rule of Five do not necessarily preclude drug development, as several clinically successful drugs, including cyclosporine A, tacrolimus, sirolimus (rapamycin), everolimus, paclitaxel, vancomycin, rifampicin, bleomycin, ritonavir, and lopinavir, are well-known examples of effective therapeutics that violate these guidelines. Accordingly, the current ADMET analysis is intended as a preliminary assessment rather than a definitive indicator of developability.

In summary, computational evidence indicates that a single small molecule can engage both the pironetin and todalam sites, offering a new route to dual-site microtubule inhibition. Compounds **4** and **8** provide tractable starting points for experimental exploration of this strategy, which may yield antimitotic agents with improved efficacy and a reduced propensity for resistance.

## 4. Materials and Methods

### 4.1. Ligand Preparation and Docking

The potential hybrid compounds were initially constructed based on the 3D structure of pironetin, obtained from the crystal structure in PDB entry 5LA6, using its characteristic three-dimensional features as a template for virtual ligand design. The designed hybrid ligands were not intended as direct synthetic targets but rather as proof-of-concept scaffolds to explore the structural feasibility of dual-site engagement. Their molecular geometries were subsequently optimized using Spartan software v.24 with the Merck Molecular Force Field (MMFF) [41]. The optimized conformations showed no significant steric clashes or highly strained features, supporting the internal structural consistency of the proposed scaffolds. SeeSAR 14.1 (BioSolveIT) software was utilized for docking studies due to its dedicated module for covalent docking and its ability to evaluate the contribution of individual ligand fragments to interactions within the binding site [42]. For molecular docking simulations, the crystal structure of the α/β-subunit of Bos taurus tubulin (PDB: 5LA6) was selected as the target. These subunits were chosen for further simulations because todalam binds at the interface between the α and β subunits, although pironetin binds exclusively to the α-subunit. The predicted dual binding site was defined based on amino acid residues previously identified as part of the pironetin binding site (33 residues) and was further expanded to include additional residues that interact with todalam within both the α- and β-subunits, resulting in a total of 54 amino acid residues. Covalent docking was performed using SeeSAR 14.1 (BioSolveIT), employing an α,β-unsaturated lactone moiety as the electrophilic “warhead” designed to undergo Michael addition with the thiol group of Cys316 in α-tubulin [22]. In SeeSAR, Cys316 was specified as the reactive center (covalent attachment site), and each ligand was placed in a pre-reactive conformation corresponding to its geometry prior to covalent bond formation. SeeSAR’s covalent docking algorithm automatically recognizes such warheads and positions them for covalent binding, accounting for the reaction’s stereochemistry and utilizing built-in covalent mechanism templates to guide correct bond formation. Covalent docking was performed with explicit definition of Cys316 as the reactive nucleophile, and the corresponding reaction mechanism (Michael addition or nucleophilic substitution, as appropriate) was specified to ensure chemically consistent bond formation. This setup enabled proper orientation of the electrophilic warhead and realistic modeling of covalent attachment at the pironetin site. For each compound, a minimum of ten distinct binding poses were generated by varying the conformations of the protein’s active site residues. The generation of these poses was guided by an incremental construction algorithm. Each pose was subsequently evaluated based on predicted binding affinity, torsional strain, steric clashes, and geometric optimization to identify the most favorable binding configuration. Since SeeSAR reports binding affinity as an interval with upper and lower boundaries, and for covalent inhibitors only the upper boundary is defined, only the upper boundary values are presented for all ligands. Binding affinity values (upper boundary) were obtained from the final SDF file generated for each ligand and were reported in micromolar (µM) units. It is important to note that the affinity evaluation considered only the non-covalent components of the ligand–protein interaction, including hydrogen bonding, hydrophobic interactions, electrostatic forces, van der Waals interactions, and related non-covalent effects. Visualization and interpretation of the obtained results were performed using UCSF Chimera 1.17.3 and Discovery Studio Visualizer v21.1.0.20298.

### 4.2. Molecular Dynamics Simulations

All-atom molecular dynamics (MD) simulations were carried out using Desmond software (Schrödinger Release 2025-4) [43] for a total simulation time of 200 ns in triplicates. Each ligand–receptor complex was solvated in an explicit water box using the TIP4P water model, and the OPLS4 force field was applied to all atoms. During the docking stage, the ligand was covalently attached to Cys316 (forming an S–C bond), and this pre-formed covalently bound complex was used as the starting structure for the MD simulations. All bonded terms (bonds, angles, dihedrals) and non-bonded interactions, including those involving the S–C linkage between the ligand and Cys316, were automatically parameterized according to OPLS4’s built-in parameterization. Consequently, no additional custom parameterization of the covalent ligand–Cys316 complex was required for the MD simulations.

To mimic physiological conditions, NaCl was added to achieve a final ionic strength of 0.15 M. Simulations were run with a time step of 2 fs, and trajectory frames were saved every 10 ps. The NPAT ensemble was employed, with temperature controlled by the Nosé–Hoover chain thermostat (relaxation time: 1.0 ps) and pressure regulated by the Martyna–Tobias–Klein barostat (relaxation time: 1.0 ps). The simulation workflow followed the default MD protocol provided by Desmond. Resulting trajectories were clustered into five groups based on ligand identity using the trajectory analysis tools available in the Schrödinger Suite. Standard parameters assessing system stability and dynamics, such as root-mean-square deviation (RMSD) and radius of gyration, were calculated from the simulation data.

## 5. Conclusions

In this study, a previously unrecognized “hybrid” binding pocket—bridging the covalent pironetin site and the adjacent todalam site on α-tubulin—was proposed and computationally validated. Covalent docking of eight in silico-designed chimeric ligands demonstrated that all derivatives can simultaneously engage the two subsites, thereby confirming the geometric and pharmacophoric continuity of the merged cavity. Among them, ligands **4** and **8** achieved submicromolar HYDE upper-boundary scores and established an extensive network of hydrogen bonds, hydrophobic contacts, and π-stacking interactions across the entire interface, despite their larger size relative to the parent ligands.

The 200 ns all-atom molecular dynamics simulations further corroborated these findings: both hybrids maintained low ligand RMSD (~1.5 Å) and compact radii of gyration, indicating persistent, well-fitted binding throughout the trajectory, whereas the remaining analogues underwent partial egress or conformational drift. The superposition of the equilibrated poses with crystallographic coordinates of pironetin and todalam confirmed accurate pharmacophoric mimicry and steric complementarity to the composite pocket.

Collectively, these results provide an in silico proof of concept that dual-site covalent inhibitors can be engineered to exploit the contiguous pironetin–todalam region of α-tubulin. Such hybrids may combine the irreversible anchoring of pironetin with the steric “molecular plug” effect of todalam and could therefore suppress microtubule assembly while simultaneously reducing the likelihood of resistance arising from single-site mutations.

This study is limited by its purely theoretical nature; predicted affinities for covalent inhibitors remain approximate, and no synthetic or biological data are yet available. Future efforts will focus on the synthetic accessibility of the top-ranked scaffolds, free-energy perturbation calculations to refine affinity estimates, and comprehensive in vitro assays, ranging from mass-spectrometric binding confirmation to antiproliferative screens in drug-resistant cancer cell lines. High-resolution structural studies by X-ray crystallography or cryo-EM of ligand–tubulin complexes will be essential to ascertain the exact binding mode and to guide further lead optimization.

## Figures and Tables

**Table 1:** Upper estimated affinity of virtual ligands.

Compound	BiosolveIT.HYDE Estimated Affinity Upper Boundary, µM
**1**	281.292
**2**	626.684
**3**	58
**4**	1.528
**5**	218,820
**6**	50.41
**7**	128.187
**8**	17.604
Pironetin	5.633
Todalam	0.254

## Data Availability

All structural coordinates used in this study were retrieved from the RCSB Protein Data Bank (PDB; https://www.rcsb.org) accessed on 30 May 2025; the corresponding accession codes are listed in the Materials and Methods section. Ligand structures and target annotations were obtained from the PubChem 2025 release (https://pubchem.ncbi.nlm.nih.gov; accessed on 30 May 2025). No new experimental data were generated. Simulation input files, processed trajectories, and any additional datasets that support the findings of this work are available from the corresponding author upon reasonable request.

## Abbreviations

The following abbreviations are used in this manuscript:

cryo EM	Cryogenic electron microscopy.
HYDE	HYdrogen bond and DEsolvation (scoring function).
MD	Molecular dynamics.
TIP4P	Transferable Intermolecular Potential, 4-Point water model.
OPLS4	Optimized Potentials for Liquid Simulations 4.
NPAT	Normal Pressure, Lateral Area, and Temperature.
SD	Standard deviation.

## References

[1] (2020). European Cancer Information System. https://Ecis.Jrc.Ec.Europa.Eu/.

[2] Bray F., Laversanne M., Sung H., Ferlay J., Siegel R.L., Soerjomataram I., Jemal A. (2024). Global cancer statistics 2022: GLOBOCAN estimates of incidence and mortality worldwide for 36 cancers in 185 countries. CA Cancer J. Clin..

[3] Sung H., Ferlay J., Siegel R.L., Laversanne M., Soerjomataram I., Jemal A., Bray F. (2021). Global Cancer Statistics 2020: GLOBOCAN Estimates of Incidence and Mortality Worldwide for 36 Cancers in 185 Countries. CA Cancer J. Clin..

[4] Tan S., Li D., Zhu X. (2020). Cancer Immunotherapy: Pros, Cons and Beyond. Biomed. Pharmacother..

[5] Zhong L., Li Y., Xiong L., Wang W., Wu M., Yuan T., Yang W., Tian C., Miao Z., Wang T. (2021). Small Molecules in Targeted Cancer Therapy: Advances, Challenges, and Future Perspectives. Signal Transduct. Target Ther..

[6] Arnst K.E., Banerjee S., Chen H., Deng S., Hwang D., Li W., Miller D.D. (2019). Current Advances of Tubulin Inhibitors as Dual Acting Small Molecules for Cancer Therapy. Med. Res. Rev..

[7] Kaur R., Kaur G., Gill R.K., Soni R., Bariwal J. (2014). Recent Developments in Tubulin Polymerization Inhibitors: An Overview. Eur. J. Med. Chem..

[8] Lafanechère L. (2022). The microtubule cytoskeleton: An old validated target for novel therapeutic drugs. Front. Pharmacol..

[9] Nepali K., Ojha R., Lee H.-Y., Liou J.-P. (2016). Early Investigational Tubulin Inhibitors as Novel Cancer Therapeutics. Expert Opin. Investig. Drugs.

[10] Hadfield J.A., Ducki S., Hirst N., McGown A.T. (2003). Tubulin and microtubules as targets for anticancer drugs. Prog. Cell Cycle Res..

[11] Naaz F., Haider M.R., Shafi S., Yar M.S. (2019). Anti-Tubulin Agents of Natural Origin: Targeting Taxol, Vinca, and Colchicine Binding Domains. Eur. J. Med. Chem..

[12] Steinmetz M.O., Prota A.E. (2024). Structure-Based Discovery and Rational Design of Microtubule-Targeting Agents. Curr. Opin. Struct. Biol..

[13] Pérez-Peña H., Abel A.-C., Shevelev M., Prota A.E., Pieraccini S., Horvath D. (2023). Computational Approaches to the Rational Design of Tubulin-Targeting Agents. Biomolecules.

[14] Alpízar-Pedraza D., Veulens A.d.l.N., Araujo E.C., Piloto-Ferrer J., Sánchez-Lamar Á. (2022). Microtubules Destabilizing Agents Binding Sites in Tubulin. J. Mol. Struct..

[15] Mühlethaler T., Gioia D., Prota A.E., Sharpe M.E., Cavalli A., Steinmetz M.O. (2021). Comprehensive Analysis of Binding Sites in Tubulin. Angew. Chem..

[16] Krause W. (2019). Resistance to Anti-Tubulin Agents: From Vinca Alkaloids to Epothilones. Cancer Drug Resist..

[17] Podolak M., Holota S., Deyak Y., Dziduch K., Dudchak R., Wujec M., Bielawski K., Lesyk R., Bielawska A. (2024). Tubulin Inhibitors. Selected Scaffolds and Main Trends in the Design of Novel Anticancer and Antiparasitic Agents. Bioorg. Chem..

[18] Shuai W., Wang G., Zhang Y., Bu F., Zhang S., Miller D.D., Li W., Ouyang L., Wang Y. (2021). Recent Progress on Tubulin Inhibitors with Dual Targeting Capabilities for Cancer Therapy. J. Med. Chem..

[19] Haider K., Rahaman S., Yar M.S., Kamal A. (2019). Tubulin Inhibitors as Novel Anticancer Agents: An Overview on Patents (2013–2018). Expert Opin. Ther. Pat..

[20] Tanabe K. (2017). Microtubule Depolymerization by Kinase Inhibitors: Unexpected Findings of Dual Inhibitors. Int. J. Mol. Sci..

[21] Tran C., Hamze A. (2025). Recent Advancements in the Development of HDAC/Tubulin Dual-Targeting Inhibitors. Pharmaceuticals.

[22] Yang J., Wang Y., Wang T., Jiang J., Botting C.H., Liu H., Chen Q., Yang J., Naismith J.H., Zhu X. (2016). Pironetin Reacts Covalently with Cysteine-316 of α-Tubulin to Destabilize Microtubule. Nat. Commun..

[23] Kondoh M., Usui T., Kobayashi S., Tsuchiya K., Nishikawa K., Nishikiori T., Mayumi T., Osada H. (1998). Cell Cycle Arrest and Antitumor Activity of Pironetin and Its Derivatives. Cancer Lett..

[24] Vogt A., McPherson P.A., Shen X., Balachandran R., Zhu G., Raccor B.S., Nelson S.G., Tsang M., Day B.W. (2009). High-Content Analysis of Cancer-Cell-Specific Apoptosis and Inhibition of in Vivo Angiogenesis by Synthetic (−)-Pironetin and Analogs. Chem. Biol. Drug Des..

[25] Mühlethaler T., Milanos L., Ortega J.A., Blum T.B., Gioia D., Roy B., Prota A.E., Cavalli A., Steinmetz M.O. (2022). Rational Design of a Novel Tubulin Inhibitor with a Unique Mechanism of Action. Angew. Chem..

[26] Fu R.G., Sun Y., Sheng W.B., Liao D.F. (2017). Designing Multi-Targeted Agents: An Emerging Anticancer Drug Discovery Paradigm. Eur. J. Med. Chem..

[27] Raghavendra N.M., Pingili D., Kadasi S., Mettu A., Prasad S.V.U.M. (2018). Dual or Multi-Targeting Inhibitors: The next Generation Anticancer Agents. Eur. J. Med. Chem..

[28] Yang J., Yu Y., Li Y., Yan W., Ye H., Niu L., Tang M., Wang Z., Yang Z., Pei H. (2021). Cevipabulin-Tubulin Complex Reveals a Novel Agent Binding Site on α-Tubulin with Tubulin Degradation Effect. Sci. Adv..

[29] Li Y., Zhang C., Tang D., Wang T., Yan W., Yang L., Bai P., Tang M., Pei H., Chen L. (2025). Identification of a Ligand-Binding Site on Tubulin Mediating the Tubulin–RB3 Interaction. Proc. Natl. Acad. Sci. USA.

[30] Talevi A. (2015). Multi-Target Pharmacology: Possibilities and Limitations of the “Skeleton Key Approach” from a Medicinal Chemist Perspective. Front. Pharmacol..

[31] Bozic I., Reiter J.G., Allen B., Antal T., Chatterjee K., Shah P., Moon Y.S., Yaqubie A., Kelly N., Le D.T. (2013). Evolutionary Dynamics of Cancer in Response to Targeted Combination Therapy. eLife.

[32] Feng J., Zheng Y., Ma W., Ihsan A., Hao H., Cheng G., Wang X. (2023). Multitarget Antibacterial Drugs: An Effective Strategy to Combat Bacterial Resistance. Pharmacol. Ther..

[33] Jackson P.A., Widen J.C., Harki D.A., Brummond K.M. (2017). Covalent Modifiers: A Chemical Perspective on the Reactivity of α,β-Unsaturated Carbonyls with Thiols via Hetero-Michael Addition Reactions. J. Med. Chem..

[34] Boike L., Henning N.J., Nomura D.K. (2022). Advances in Covalent Drug Discovery. Nat. Rev. Drug Discov..

[35] Prota A.E., Setter J., Waight A.B., Bargsten K., Murga J., Diaz J.F., Steinmetz M.O. (2016). Pironetin Binds Covalently to ACys316 and Perturbs a Major Loop and Helix of α-Tubulin to Inhibit Microtubule Formation. J. Mol. Biol..

[36] Araki M., Matsumoto S., Bekker G.-J., Isaka Y., Sagae Y., Kamiya N., Okuno Y. (2021). Exploring Ligand Binding Pathways on Proteins Using Hypersound-Accelerated Molecular Dynamics. Nat. Commun..

[37] Takasawa T., Matsui T., Watanabe G., Kodera Y. (2024). Molecular Dynamics Simulations Reveal Differences in the Conformational Stability of FtsZs Derived from Staphylococcus Aureus and Bacillus Subtilis. Sci. Rep..

[38] Chattopadhyaya S., Chakravorty D., Basu G. (2019). A Collective Motion Description of Tubulin ΒT7 Loop Dynamics. Biophys. Physicobiol..

[39] Awoonor-Williams E., Abu-Saleh A.A.-A.A. (2021). Covalent and Non-Covalent Binding Free Energy Calculations for Peptidomimetic Inhibitors of SARS-CoV-2 Main Protease. Phys. Chem. Chem. Phys..

[40] Homeyer N., Gohlke H. (2012). Free Energy Calculations by the Molecular Mechanics Poisson−Boltzmann Surface Area Method. Mol. Inform..

[41] Halgren T.A. (1999). MMFF VII. Characterization of MMFF94, MMFF94s, and Other Widely Available Force Fields for Conformational Energies and for Intermolecular-Interaction Energies and Geometries. J. Comput. Chem..

[42] (2025). SeeSAR.

[43] Li J., Abel R., Zhu K., Cao Y., Zhao S., Friesner R.A. (2011). The VSGB 2.0 Model: A next Generation Energy Model for High Resolution Protein Structure Modeling. Proteins Struct. Funct. Bioinform..

